# Clinical evaluation of direct anterior approach total hip arthroplasty for severe developmental dysplasia of the hip

**DOI:** 10.1038/s41598-021-87543-x

**Published:** 2021-04-14

**Authors:** Zaiyang Liu, Courtney D. Bell, Alvin C. Ong, Jun Zhang, Jie Li, Yuan Zhang

**Affiliations:** 1 Department of Orthopedics, Joint Disease and Sport Medicine Center, Xinqiao Hospital, Army Medical University, 183# Xinqiao Street, Shapingba District, Chongqing, 400037 China; 2 Rothman Orthopaedic Institute, Building 1300, 2500 English Creek Ave,, Egg Harbor Township, NJ 08234 USA

**Keywords:** Outcomes research, Bone

## Abstract

It is challenging to treat developmental dysplasia of the hip (DDH) classified Crowe III-IV using direct anterior approach (DAA) total hip arthroplasty (THA), and very little is known on its outcome. This study aimed to investigate the clinical result in this defined disorder with DAA versus posterolateral approach. Twenty-three consecutive hips with Crowe III-IV DDH who underwent DAA were retrospectively evaluated from 2016 through 2018. Outcomes were primarily assessed by HHS, WOMAC, and SF-12 physical scales. The second evaluations included leg length discrepancy, hip muscle strength, radiographic review, complications, and limp recovery. Results were compared to a control cohort of 50 hips underwent posterolateral THA concurrently within the observational period. At last follow-up (DAA 28.5 months; PLA 39.0 months), the mean increase of the HHS for DAA was 48.2 and 30.3 for PLA (p = 0.003). The improvement in WOMAC score in DAA cohort was 15.89 higher that of the PLA cohort after adjusting preoperative difference [R2 = 0.532, P = 0.000, 95% CI (10.037, 21.735)]. DAA had more rapid recovery of hip abductor strength at 1-month (p = 0.03) and hip flexor strength at 3 months (p = 0.007) compared to PLA. No significant differences were found in the radiographic analysis with the exception of increased acetabular anteversion in the DAA cohort (p = 0.036). Satisfactory improvement in limp, indicated by the percentage of limp graded as none and mild to the total, was much higher in DAA cohort (97.6%), compared to that of PLA cohort (90.0%, p = 0.032). DAA for high-dislocated dysplasia demonstrate a significant improvement in clinical result comparable to posterolateral approach. Improved clinical outcome in terms of increased HHS and WOMAC scores, rapid recovery of hip abductor and flexor strength, and enhanced limp recovery without an increased risk in complications, could be acquired when the surgeons were specialized in this approach.

## Introduction

There continues to be a rising interest in using direct anterior approach (DAA) in total hip arthroplasty (THA), despite the challenges of transitioning from traditional approaches^1^. The DAA is generally considered for simple and primary hip disorders such as avascular necrosis, osteoarthritis, femoral neck fractures, and Crowe type I–II developmental dysplasia of the hip (DDH)^2^. High-dislocated DDH, defined as type III–IV in the Crowe system^3^, is not typically treated with the DAA due to the technically challenging surgery and complex pathology^4–7^.

There is a large population of patients with severe DDH in the senior author’s (Y.Z.) practice region of Southwest China with a high demand for DAA surgery. At the senior author’s hospital, over 2000 cases of osteoarthritis have been treated with THA via the DAA since 2015, including over 300 cases of DDH of all levels of severity. The current literature is limited to small case series of DDH patients who have undergone THA via the DAA^8–10^. Historically, the literature has focused on surgical management of Crowe III–IV dysplasia with THA via the posterolateral (PLA), anterolateral, or lateral approaches^11,12^.

Our hypothesis was that THA via DAA for patients with Crowe type III-IV dysplasia improves clinical outcomes, has a low rate of complications, and has comparable outcomes to the PLA. The results of this study should help guide the surgeon with DAA experience on the best choice of management for these complex disorders.

## Methods

This is a retrospective and observational study of all DAA primary THAs completed by a single surgeon (Y.Z.) for the diagnosis of osteoarthritis secondary to Crowe III–IV DDH. All the dislocated hips including bilateral DDH were treated with one-stage DAA or PLA THAs in this series. Exclusion criteria included: Bilateral DDH but treated with unilateral THA, possible infection around hip, severe contracture of the hip caused by previous surgery, DDH combined with a neurovascular disease and poliomyelitis. Consent form from each patient were also acquired (All patients agreed to use their data for study and publication and there is no patients under the age of 18 years) and approved by the ethics committee (Health Center Institutional Review Board, Xinqiao Hospital, Arm medical university).All of the medthods used were strictly compliance with DDH treatment guidelines.

### Data source

A total of 275 patients diagnosed with osteoarthritis secondary to DDH classified from type I to type IV in Crowe system, were obtained from the author’s institutional database from Jan. 1st, 2016 through Jan. 1st, 2018. The observational end of this study was Mar. 1st, 2020. Of these patients, 69 patients were classified into Crowe Type III–IV. Twenty-two patients (23 hips) out of the 69 patients underwent DAA THA was set as the observational cohort, compared with the remaining 47 patients (50 hips) treated with PLA THA concurrently within the observational period as the control cohort. The assignment of patients to one specific approach was determined by patients’ decision under formal and adequate notification by chief surgeon, regarding benefit and risk of the approaches. Patients’ demographical characteristics and radiographs were reviewed and medical documents of follow-up were retrieved to evaluate component positioning and clinical outcomes.

### Surgical technique

**Figure 1:**
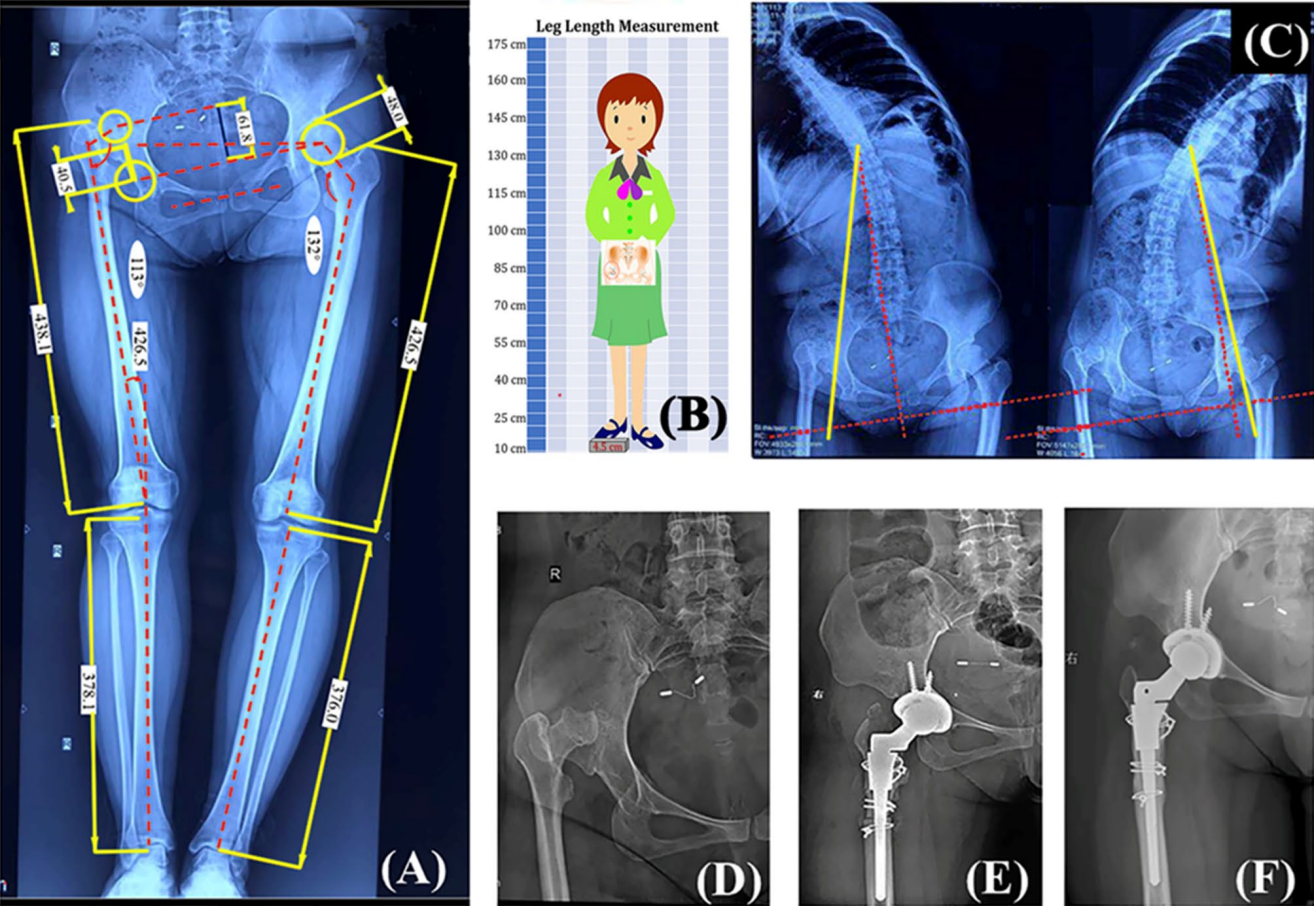
Preoperative planning and surgical treatment of a 37-year-old male patient diagnosed as Crowe type IV DDH by DAA THA combining STO. (**A**) Preoperative planning and lower limb length balancing by standing full-length anteroposterior radiographs. A stepwise algorithm for limb length equalization was implemented based on measurements of the hip dislocation height, the anatomical lengths of the lower limbs, the pelvic tilt angle, intra-articular and extra-articular deformities as indicated. (**B**) The limb length discrepancy was further evaluated by a scaled block under the ipsilateral foot in our measuring system. (**C**) Bilateral bending X-ray of lumbar spine were obtained to estimate the flexibility of the lumbar-pelvic complex. (**D**,**E**) Preoperative and postoperative anteroposterior hip radiographs. This patient was further balanced by STO within direct anterior approach (osteotomy length, 25 mm). (**F**) Osteotomy union at 7 months after surgery.

**Figure 2:**
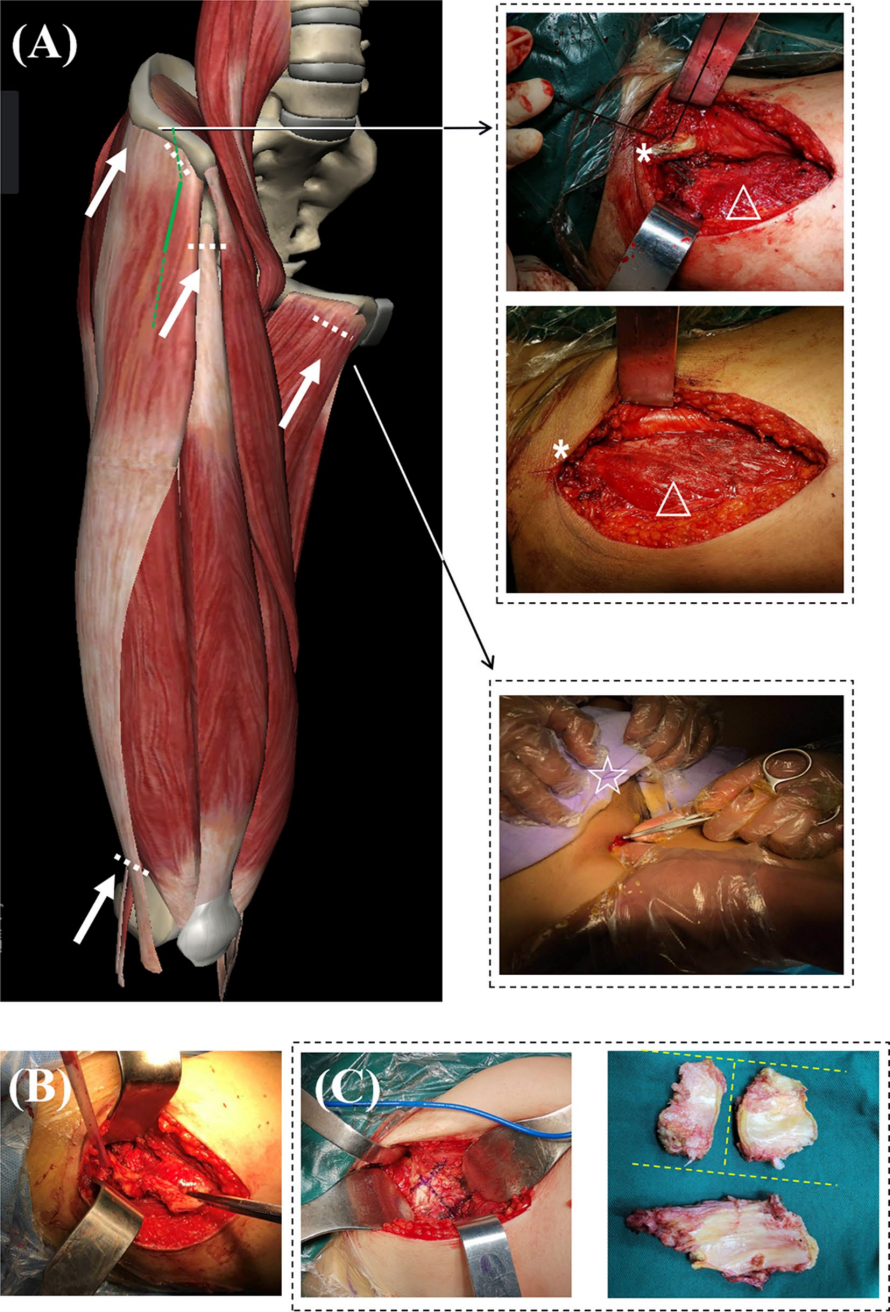
Soft tissue balancing techniques for Crowe III-IV DDH in DAA THA. (**A**) Common structures for soft tissue release include the tensor fascia latae (TFL), adductor tendon, rectus femoris, and distal iliotibial band (white arrows). A standard DAA (full green line) can be converted into an extensile DAA (dotted green line) when further extension was required. The upper right represents peel-off technique of TFL at the iliac attachment and repair technique using non-absorbable sutures (* anterior superior iliac spine, ∆ TFL belly.) The middle right represents pie-crusting technique of the adduction tendon (☆ symphysis pubis). (**B**) Resection of whole capsule in a sleeve-like en-bloc capsulectomy technique. (**C**) Anterior capsule resection in a H-shape manner after the Hueter interval is fully obtained. Resected anterior and posterior capsule.

Preoperative planning was completed with standard anteroposterior and lateral hip radiographs as well as full-length weight-bearing and lumbar radiographs (Fig. 1). A standard DAA as described by Post and York was initiated on a standard operating room table^2,13^. Appropriate extension were considered to obtain an extensile exposure for difficult cases. Initial release was obtained by resecting the whole capsule in a sleeve-like en-bloc capsulectomy technique (Fig. 2B,C). Soft tissue contractures were addressed with peeling-off the tensor fasciae latae (TFL), partial release of tight structures as indicated (psoas tendon, iliotibial band, reflected head of the rectus, adductor tendon) (Fig. 2A). The dysplastic acetabulum was reconstructed by either structural bone autograft or porous metal augment^14^. And the acetabular component was placed at the true hip center regardless of deformity. A transverse subtrochanteric osteotomy (STO) was indicated for anatomic or functional equalization of the lower limb depending on the compensatory mechanism of the pelvic and lumbar complex^15^, or to prevent sciatic nerve injury secondary to over-lengthening of the leg (Fig. 1). The specific methods in soft tissue release and limb length equalization were reported in our previous publication^14^.

### Radiographic review

Radiographs were reviewed and measured by an independent researcher blinded to the research protocol, by evaluating the component positioning and were compared to the control cohort. Measurements recorded were acetabular inclination, acetabular anteversion, stem subsidence, and rotation center deviation.

### Outcome measures

Clinical follow-up was performed at 1, 3, 6, and 12 months after the surgery and annually thereafter. The primary evaluations of patient-reported outcome including Harris Hip Score (HHS), Western Ontario and McMaster Universities Osteoarthritis Index (WOMAC), and Short-Form 12-item Health Survey (SF-12), which were recorded from preoperative to the last follow-up. Secondary outcome measures were leg length discrepancy (LLD), hip abductor and flexor strength, radiographic analysis, limp recovery, and postoperative complications. Muscle strength of hip abductor and flexor was determined in lateral decubitus position and seated position, respectively. The score was recorded from 0 to 5 according to the Oxford Scale, by performing manual or gravitational resistance to hip abduction and flexion. Hip abduction or flexion angle more than 60 degree under manual resistance or gravity was considered as positive for scale V or IV. The recovery of limp was classified into none (normal gait), mild (tolerable), moderate (positive Trendelenburg sign) and severe (walking disability), as used in most studies^16^. Limp in none and mild grades were considered as satisfactory in outcome assessment. Both of the muscle strength and limp evaluation were conducted by an independent physical therapist in our clinic, who was blinded to the research protocol.

### Statistical analysis

This study was designed and reported by STROBE criteria^17^, All the data were analyzed using SPSS software, version 23.0 (IBM, USA). T-tests were used to analyze continuous variables, Chi-square or Fisher’s Exact testing were used for categorical variables, and Wilcoxon rank sum test were employed for ranked variables. A linear regression model was used to analyze the difference of WOMAC score. A p-value of < 0.05 was considered significant.

## Results

### Preliminary comparison

Demographic variables including age, gender, BMI, LLD and limp severity did not differ between cohorts (p > 0.05, Table 1). Side of THA did differ between the two cohorts (p = 0.015). There was no significant difference between the two cohorts in terms of the ratios of the bilateral operation or previous hip surgery to all the surgeries. Radiographical measurement indicated no significant difference regarding Crowe type classification (p = 0.910), central-edge angle (p = 0.081), dislocation height of the femoral head to the true acetabulum (p = 0.154), and the LLD caused by pelvic tilt and lumbar inclination, and bony length of the femur and tibia.

**Table 1:** Baseline characteristics in demographic and preoperative assessments of the two study cohorts.

Indicators	DAA (22 patients 23 hips)	PLA (47 patients 50 hips)	p-value
Age (years)	42.0 (13.6)	47.8 (17.6)	0.262
BMI	21.9 (3.2)	23.5 (4.3)	0.460
**Gender**			
Female	18 (81.8%)	33 (70.2%)	0.445
Male	4 (18.2%)	14 (29.8%)	
**Crowe classification**			
Type III	10 (43.5%)	25 (50.0%)	0.910
Type IV	13 (56.5%)	25 (50.0%)	
**Operational side**			
Left	15 (65.2%)	13 (26.0%)	0.015
Right	8 (34.8%)	37 (74.0%)	
Bilateral Surgery	1	3	0.761
Previous hip surgery	0	2	0.326
Central-edge angle (°)	− 13.84 (22.84)	− 4.92 (15.25)	0.081
Dislocation height (mm)	44.8 (19.3)	52.5 (23.6)	0.154
Leg length discrepancy (mm)	22.1 (13.7)	31.0 (16.8)	0.113
**Limp**			
Mild	1 (4.5%)	2 (4.3%)	0.069
Moderate	8 (36.4%)	7 (14.9%)	
Severe	13 (59.1%)	38 (80.9%)	

### Operative variables

The mean operative time for DAA was 149.6 min (SD 62.6 min) and 128.5 min (SD 50.8 min) for PLA, from incision to closure (p = 0.305). The mean estimated blood loss (EBL) for DAA was 584.6 ml (SD 157.3 ml) and 434.6 ml (SD 257.2 ml) for PLA (p = 0.031). Blood transfusion was indicated when the intraoperative blood loss over 800 ml, or intraoperative hemoglobin less than 90 g/ml by blood test, or the operative time over 120 min. And the overall transfusion rate was 53.8% for both cohorts. The adjuvant procedures during surgery included, 10 hips (43.5%) in the DAA cohort, and 24 hips (48.0%) in the PLA cohort required transverse and shortening STO (p = 0.267) with a mean length of 2.33 cm (range 1.5–3 cm; SD 0.76 cm) and 1.71 cm (range 0.5–3 cm; SD 0.86 cm), respectively (p = 0.317). 7 hips (30.4%) in DAA cohort underwent peeling-off the TFL, while 15 hips (30.0%) in PLA cohort underwent peeling-off the gluteus maximus (GM), to reach extensile direct anterior or posterolateral approach. The detached tendons of TFL and GM were repaired anatomically by anchors or trans-tunnel technique. Prosthesis profile showed that the implant parameters of the two cohort exhibited no statistical difference (Table 2).

**Table 2:** Implants parameters of the two study cohorts.

Indicators	Direct anterior (n = 23)	Posterolateral (n = 50)	p-value
**Bearing surface**			
Ceramic on ceramic	18 (78.3%)	33 (66.0%)	0.721
Ceramic on polyethylene	5 (21.7%)	17 (34.0%)	
**Head size**			
22 mm	0 (0.0%)	1 (2.0%)	0.915
28 mm	19 (82.6%)	42 (84.0%)	
32 mm	4 (17.4%)	7 (14.0%)	
**Cup size**			
38 (mm)	0 (0.0%)	2 (4.0%)	0.780
44 (mm)	15 (65.2%)	32 (64.0%)	
46 (mm)	4 (17.4%)	9 (18.0%)	
48 (mm)	3 (13.0%)	4 (8.0%)	
50 (mm)	1 (4.3%)	3 (6.0%)	
**Femoral stem**			
S-ROM	22 (95.7%)	47 (94.0%)	0.925
Trilock	1 (4.3%)	0 (0.0%)	
Corail	0 (0.00%)	3 (6.0%)	
**Stem size**			
7 (S-ROM)	0 (0.0%)	7 (14.0)	0.842
8 (S-ROM)	8 (34.8)	12 (24.0)	
9 (S-ROM)	11 (47.8)	18 (36.0)	
11 (S-ROM)	3 (13.0)	7 (14.0)	
13 (S-ROM)	0 (0.0%)	3 (6.0%)	
1 (Trilock)	1 (4.3%)	0 (0.0%)	
6 (Corail)	0 (0.0%)	1 (2.0%)	
7 (Corail)	0 (0.0%)	2 (4.0%)	

### Clinical outcomes

**Figure 3:**
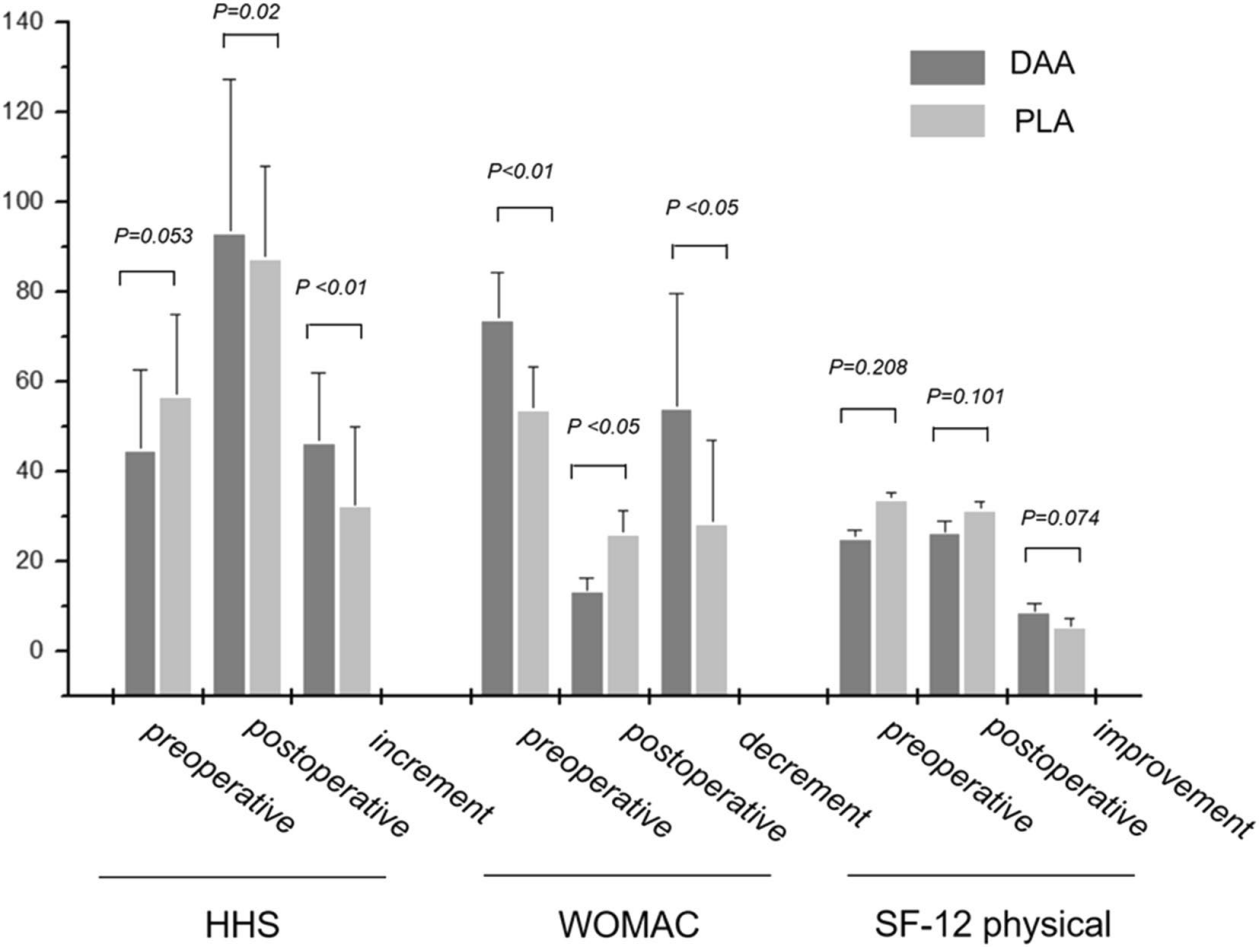
The primary evaluations by patient-reported outcomes of HHS, WOMAC, and SF-12 (physical) measures. Each measure was expressed by preoperative, postoperative values and changes in increment or decrement. P < 0.05 was considered as statistically significant.

All the patients in the two cohorts engaged in the clinical follow-up program. The average follow-up period was 28.5 months (range 26–45 months) for the DAA cohort and 39.0 months (range 26–48 months) for the PLA cohort. The mean change of the HHS from preoperative to last postoperative follow-up for the DAA cohort was 48.2 (range 28–73; SD 15.5) and 30.3 (range 11–67; SD 17.6) for the PLA cohort (p = 0.003). The postoperative scores for both PLA and the DAA cohorts achieved above a minimal clinically important difference for the HHS^18^, which is 7–9, and had a statistically significant increase (p < 0.001). In terms of WOMAC assessment, the preoperative baseline of the two cohorts was obviously different (p < 0.001), which might result in bias in comparing the postoperative outcome. Hereby we employed a linear regression to adjust the preoperative discrepancy, and the results indicated that postoperative score remained significantly different [R2 = 0.532, p = 0.000, 95% CI (10.037, 21.735)]. The average score in DAA cohort was 13.5, which was lower than that of the PLA cohort (26.2). The finding in the improvement of SF-12 physical score was non-significant at the final follow-up (p < 0.074, Fig. 3).

The mean LLD preoperatively was 3.21 cm (SD 1.37 cm) for the DAA cohort and 3.10 cm (SD 1.68 cm) for the PLA cohort preoperatively (p = 0.198). The LLD reduced to a mean of 0.2 cm (SD 0.4 cm) for the DAA cohort and 0.4 cm (SD 0.7 cm) for the PLA cohort at the last clinic follow-up (p = 0.390).

Hip abductor and flexor strength did not differ between the two cohorts preoperatively. Both DAA and PLA cohorts had a significant increase in hip abductor strength from preoperative to 1-month (DAA p = 0.030; PLA p < 0.001) and 3 months postoperatively (DAA p = 0.001; PLA p < 0.001). At 1-month postoperative, the DAA had a more rapid recovery in abductor strength as compared to the PLA (p = 0.03) with no significant difference at 3 months postoperative (p = 0.252). For hip flexor strength, there were no significant changes in the DAA cohort. The PLA cohort had a significant decrease in hip flexor strength at 1-month postoperative as compared to preoperative (p < 0.001) with no significant difference at 3 months postoperative (p = 0.279). At 3 months postoperative, the DAA had significantly higher hip flexion strength as compared to the PLA (p = 0.007). The differences of muscle strength recovery appeared to be negligible by the 6th month postoperatively (Table 3).

**Table 3:** Changes in hip abductor and flexor strength before and after THA in the two cohorts.

	DAA (n = 23)				PLA (n = 50)				p-value
	2	3	4	5	2	3	4	5	
**Hip abductor strength**									
Preoperative	21.7% (5)	47.8% (11)	30.4% (7)	0 (0)	26.0% (13)	50.0% (25)	24.0% (12)	0 (0)	0.911
1 month postop	0 (0)	17.3% (4)	69.6% (16)	13.1% (3)	0 (0)	46.0% (23)	54.0% (27)	0 (0)	0.030
3 months postop	0 (0)	0 (0)	39.1% (9)	60.9% (14)	0 (0)	12.0% (6)	54.0% (27)	34.0% (17)	0.252
6 months postop	0 (0)	0 (0)	4.3% (1)	95.7% (22)	0 (0)	6.0% (3)	12.0% (6)	82.0% (41)	0.605
**Hip flexor strength**									
Preoperative	0 (0)	0 (0)	43.4% (10)	56.6% (13)	0 (0)	8.0% (4)	54.0% (27)	38.0% (19)	0.550
1 month postop	0 (0)	21.7% (5)	52.2% (12)	26.1% (6)	0 (0)	60.0% (30)	32.0% (16)	8.0% (4)	0.065
3 months postop	0 (0)	0 (0)	30.4% (7)	69.6% (16)	0 (0)	20.0% (10)	62.0% (31)	18.0% (9)	0.007
6 months postop	0 (0)	0 (0)	13.0% (3)	87.0% (20)	0 (0)	0 (0)	12.0% (6)	88.0% (44)	0.754

### Radiographic analysis

Compared to the control cohort there were no significant differences in component placement on postoperative anteroposterior radiograph analysis with the exception of acetabular anteversion. Acetabular anteversion means were 15.8° for DAA (SD 3.46°) and 13.1° for PLA (SD 3.75°) (p = 0.036). Acetabular inclination means were 38.3° for DAA (SD 8.00°) and 38.0° for PLA (SD 6.08°) (p = 0.899). The rotation center deviation was 4.79 mm (SD 3.81 mm) for DAA and 4.42 mm (SD 3.15) for PLA in reference to the anatomic center (p = 0.766). The difference in femoral offsets of the operational to the contralateral side was 3.68 mm (SD 2.25 mm) for DAA and 5.13 mm (SD 3.19 mm) for PLA (p = 0.397).The mean stem subsidence for the DAA cohort was 1.41 mm (SD 1.15 mm) and 1.61 mm (SD 1.49 mm) for the PLA cohort (p = 0.647).

### Complications

No significant differences were found between the two cohorts in terms of non-specific complication including wound issues and dislocation (Table 4). This was no statistical difference regarding nerve injury (p = 0.253), the DAA cohort had 2 transient lateral femoral cutaneous nerve neuropraxias, which spontaneously recovered within 3 months. While the PLA cohort had 1 sciatic nerve neuropraxia, and the patient recovered full function with oral Vitamin B12 supplements at 8 months postoperatively. 13.0% of the patients in the DAA and 12.0% in the PLA cohort progressed to genu valgus (p = 0.310), which was resolved by supracondylar iliotibial band releases at the third month postoperatively. The DAA cohort had 2 cases of non-displaced fractures (cracks) at the distal aspect of the femoral stem recognized on postoperative radiographs. One patient subsequently fell down stairs and developed a periprosthetic femur fracture (Vancouver C), which was managed with a minimally invasive open reduction and internal fixation, and bone healing was observed at 6th month after fixation. Three cases of non-displaced distal femur fracture were recognized in the PLA cohort (p = 0.202). The PLA cohort had 2 cases of revision, both for aseptic loosening (1 for the acetabular component at the 8th month after THA and 1 for mechanical loosening of the femoral stem due to non-union at the osteotomy site at the 14th month after THA). No cases of revision were found in the DAA cohort (p = 0.305).

**Table 4:** Post-operative complications and limp recovery in the two cohorts at the final follow-up.

	DAA (22 patients 23 hips)	PLA (27 patients 50 hips)	p-value
Wound issues	5 (21.7%)	6 (12.0%)	0.425
Neuropraxia	2 (8.7%)	1 (2.0%)	0.253
Secondary genu valgus	3 (13.0%)	6 (12.0%)	0.310
Intraoperative crack	2 (8.7%)	3 (6.0%)	0.202
Periprosthetic fracture	1 (4.3%)	0	0.138
Dislocation	0 (0%)	3 (6.0%)	0.230
Revision surgery	0 (0%)	2 (4.0%)	0.305
Non-union	0 (0%)	1 (2.0%)	0.550
**Limp**			
None	10 (43.4%)	21 (44.6%)	0.704
Mild (tolerable)	11 (52.2%)	16 (34.0%)	
Moderate (trendelenburg sign)	1 (4.3%)	7 (14.8%)	
Severe (walking disability)	0 (0%)	3 (6.4%)	

Wilcoxon rank sum test revealed that limp in both DAA and PLA cohorts were significantly alleviated by surgical treatment at the final follow-up (DAA, Z = − 0.499, p < 0.001; PLA, Z = − 5.900, p < 0.001). The majority of patients in both cohorts had no limp (DAA 43.4%; PLA 46.0%) or a mild limp (DAA 52.2%; PLA 34.0%) postoperatively. Although there were no significant differences between the two cohorts with respect to the overall distribution of limp severity (Z = − 0.214; p = 0.831). Satisfactory improvement of limp, represented by none and mild grades in total limp demonstrated much higher percentage in DAA cohort than those in PLA cohort (Table 4). Particularly, 3 patients (6.38%) in the PLA cohort had severe postoperative limp. No obvious alleviation was found after active abductor muscle strength training over 12 months.

## Discussion

Traditional THA for Crowe type III-IV DDH typically employs a fluted modular stem or extensively porous-coated stem via a posterolateral approach. Most studies have shown satisfactory middle- to long-term clinical results^18,19^. However, some limitations in this approach have been recognized, such as invasion of the short external rotators increasing dislocation^20^, and interruption of the medial femoral circumflex branch of the femoral artery impairing osteointegration on bone-prothesis interface and bone union at the osteotomy site^21^. These deficiencies could be properly addressed by DAA as we proposed in our previous publication^22^, and this inference was fully substantiated by prior uncontrolled study^23^.

To our knowledge, there are very few studies reporting THA via the DAA in treating Crowe type III–IV DDH. Oinuma did report the early outcomes of 9 patients (12 hips) diagnosed with Crowe IV dysplasia by DAA with STO, functional improvement seemed to be satisfactory at a mean follow-up period of 3.7 years^10^. Our series demonstrates similar functional improvements, but with a decreased surgery duration and blood loss even for those patients who underwent a STO compared to this study. Another uncontrolled study reported an average 8.4-year outcomes of 23 case series treated with DAA in supine position on a traction table. The improvement of HHS and WOMAC scores were satisfactory, but three hips were revised due to wear-induced loosening at an early stage after surgery^9^.

This study presented a single surgeon series of patients treated with DAA THA for Crowe III-IV DDH, by evaluating the effectiveness and benefit-risks of DAA compared to traditional approach. We demonstrated a clinically important difference in the increase of the HHS and WOMAC when performing the surgery via a DAA with a low complication rate comparable to the PLA. Our result did have a lower rate of complications as compared to the select patients in the study by Cameron who underwent THA via the DAA for DDH^24^. We do report an increased EBL in the DAA compared to the PLA; however, this did not result in an increased rate of transfusion. In addition, despite the non-significant difference in surgical time between the two cohorts, the average increase for the DAA cohort was 21 min more than PLA cohort in our well trained and experienced surgical team. Since the chance of infection increases with surgical time^25^, careful planning and advanced practice are advised to those who may think of trying direct anterior approach in Crowe III-IV DDH, in case of increased occurrence of surgical site infection.

The DAA spares the abductor complex, which may already be weak in these patients. Kawasaki et al. found damages to the gluteus minimus, obturator internus and tensor fascia latae in the case series with DDH underwent DAA, but sparing of the gluteus medius and piriformis^26^. We demonstrated no clinically relevant injury to the hip flexors as measured by muscle strength in patients who underwent the DAA as compared patients who underwent the PLA and a more rapid improvement in hip flexor and abductor strength.

The lumbar-pelvic-hip complex also plays a key role in decision-making for Crowe type III–IV DDH^6,27^. DAA in the supine position is advantageous to reproducibly gain a functional pelvic position, which is beneficial for accurate sizing and safe positioning of the component^28,29^. As we reported in our series, both the clinical outcomes and radiographic analyses demonstrated comparable results between the DAA and the PLA.

The limitations of this study include a small sample size, retrospective but non-randomized design, and long-term clinical data are unavailable at present. This limitation needs to be addressed by conducting a multicenter, prospective cohort study with a large sample population of our undergoing work, and a randomized controlled trial (ChiCTR2100043095) for further study is on-going to explore the definite value of this technique in high-dislocated hip dysplasia.

The strengths of the study are: first, it is a single surgeon case series with consistent surgical procedure and follow-up program, which avoids the subjective bias in the comparative study performed by multiple surgeons. Second, this study also includes a precise radiographic review of the postoperative radiographs (especially rotation center deviation and stem subsidence) comparing DAA to PLA. Third, this is also the first paper to our knowledge to quantitatively measure postoperative hip abductor and flexor muscle strength after DAA in high-dislocated hip dysplasia.

## Conclusions

DAA for high-dislocated dysplasia demonstrates a significant improvement in clinical result comparable to posterolateral approach. This study demonstrated improved outcome was acquirable, in terms of increased HHS and WOMAC scores, rapid recovery of hip abductor and flexor strength, and enhanced limp recovery without an increased risk in complications.
